# The need of a ‘self-evolving’ research platform in tribology

**DOI:** 10.1093/nsr/nwag447

**Published:** 2026-07-21

**Authors:** Daniele Dini

**Affiliations:** Department of Mechanical Engineering, Imperial College London, UK

Tribology is the pervasive science of interacting surfaces in relative motion, encompassing contact, friction, wear, lubrication, adhesion and wetting [1,2]. These phenomena arise from complex interactions across multiple length-scales and timescales, from atomic and molecular processes to engineering systems operating in demanding environments. Because tribological performance depends simultaneously on material composition, surface topography, interface chemistry, operating conditions and environmental factors, optimization rapidly becomes a high-dimensional and strongly non-linear problem. Traditional trial-and-error approaches are increasingly inadequate for exploring such vast design spaces and delivering predictive, targeted solutions.

Across chemistry [3], life sciences [4] and materials engineering [5], autonomous experimentation is transforming scientific discovery. By integrating automation, machine learning and adaptive decision making within closed-loop frameworks, ‘self-evolving experiments’ can iteratively design, execute and learn from experiments, dramatically accelerating exploration of complex parameter spaces. Such approaches are particularly attractive for tribology, where competing objectives—including friction reduction, wear resistance, durability, efficiency and sustainability—must often be balanced simultaneously.

**Figure 1.**
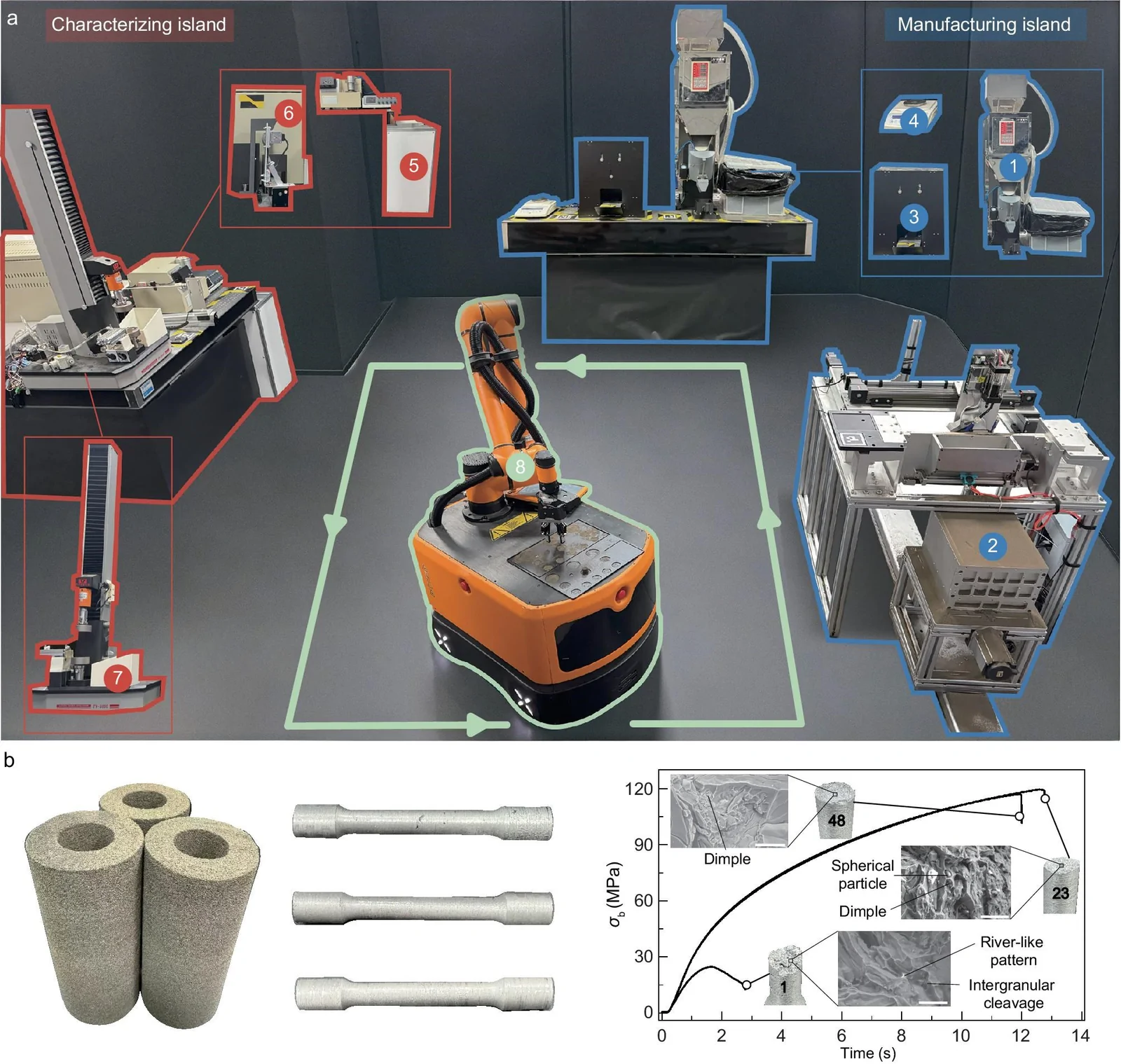
Self-evolving experimental platform Ex3DSP for 3D sand printing. (a) Ex3DSP combines a robotic platform comprising two functional islands and eight automated workstations with a multi-objective active-learning framework. (b) Application to sand mould fabrication and casting optimization. Reproduced from Hu *et al*. [6] under the terms of the Creative Commons CC BY license.

In this context, Hu and co-workers report a self-evolving experimental platform, Ex3DSP, that combines robotics and artificial intelligence to optimize 3D sand printing [6]. The platform establishes a ‘design–manufacture–characterize–learn’ loop capable of autonomously identifying optimal process conditions within a large multidimensional parameter space. Remarkably, only 60 experiments were required to map the Pareto front of a three-objective, three-variable optimization problem, reducing experimental effort by >3 orders of magnitude compared with exhaustive testing. Beyond identifying optimal parameter combinations, the platform elucidated the mechanisms linking droplet wetting, penetration, curing and bonding to the performance of printed sand moulds. Significant improvements in strength, gas evolution and permeability were achieved, ultimately yielding a 385% enhancement in casting performance (Fig. 1). This study highlights the potential of autonomous platforms to shift experimentation from empirical trial-and-error toward precise targeting.

The significance of this work extends far beyond wetting and additive manufacturing. Tribology is fundamentally a system science characterized by coupled interfacial phenomena, multiscale interactions and competing performance metrics. Friction, wear and lubrication depend on the interplay of materials, surface engineering, contact mechanics, chemistry and operating conditions, creating experimental design spaces that are often too large to explore systematically using conventional methods. Self-evolving research frameworks could accelerate the discovery of lubricants, coatings and surface textures, identify operating conditions that maximize efficiency and durability, and reveal previously hidden relationships governing interfacial behavior.

Perhaps most importantly, such approaches could help transform tribology from a largely empirical discipline into a predictive science driven by data, physics-based modeling and autonomous experimentation [7]. The integration of autonomous experimentation with multiscale simulations—from *ab initio* calculations and molecular dynamics to continuum-scale models [2,8]—offers a particularly promising route. This ‘dry–wet’ discovery framework [9] would combine the speed of computation with the fidelity of experimentation, enabling continuous exchange between virtual and physical laboratories. As tribology addresses challenges associated with electrification, renewable energy, advanced manufacturing and net-zero objectives, self-evolving platforms may not simply improve tribological research—they may redefine how discoveries in tribology are made.
